# Combined PCL instabilities cannot be identified using posterior stress radiographs in external or internal rotation: A cadaveric study

**DOI:** 10.1002/ksa.12458

**Published:** 2024-09-11

**Authors:** Thorben Briese, Romy Riemer, Adrian Deichsel, Christian Peez, Elmar Herbst, Johannes Glasbrenner, Michael J. Raschke, Christoph Kittl

**Affiliations:** ^1^ Department of Trauma, Hand and Reconstructive Surgery University Hospital Münster Germany; ^2^ Marien Hospital Soest Academic Teaching Hospital of the University of Münster Münster Germany

**Keywords:** combined posterior instability, posterior cruciate ligament, posterior tibial displacement, posterolateral corner, posteromedial corner

## Abstract

**Purpose:**

Posterior stress radiography is recommended to identify isolated or combined posterior cruciate ligament (PCL) deficiencies. The posterior drawer in internal (IR) or external rotation (ER) helps to differentiate between these combined instabilities. The purpose of this study was to evaluate posterior stress radiography (PSR) in isolated and combined PCL deficiency with IR and ER compared to PSR in neutral rotation (NR) for diagnosing combined PCL instabilities.

**Methods:**

Six paired fresh‐frozen human cadaveric legs (*n* = 12) were mounted in a Telos device for PSR. The tibia was rotated using an attached foot apparatus capable of rotating the foot 30° internally and externally. A posterior tibial load of 15 kp (147.1 N) was applied to the tibial tubercle at 90° knee flexion, and a lateral radiograph was obtained. This was repeated with the foot in 30° IR and ER. The PCL, posterolateral complex (PLC), and posteromedial complex (PMC) were sectioned in six knees, while the PMC was sectioned before the PLC in the other six knees. Posterior tibial displacement (PTD) was measured radiographically. Statistical analysis was performed using a two‐way ANOVA and a mixed model with Bonferroni correction, and the significance was set at *p* < 0.05. Furthermore, intra‐ and interobserver reliability was determined.

**Results:**

Cutting the PCL significantly increased the radiographic PTD by 9.8 ± 1.8 mm (side‐to‐side difference compared to the intact state of the knee, *n* = 12; *p* < 0.001). This further increased to 12.2 ± 2.3 mm (*n* = 6; *p* < 0.01) with an additional PLC deficiency and to 15.4 ± 3.4 mm (*n* = 6; *p* < 0.05) with an additional PMC deficiency. A combined PLC and PMC deficiency resulted in an increase of the PTD to 15.9 ± 4.5 mm (*n* = 12; *p* < 0.01). In the PCL/PLC deficient state, ER did not demonstrate a higher PTD, compared to the NR and IR posterior drawer. In the PCL/PMC deficient state in IR, PTD was 1.6 ± 0.7 mm (*p* < 0.01) higher compared to NR and 3.2 ± 1.9 mm (*p* < 0.05) higher compared to ER. We showed excellent intra‐ and interobserver reliability (0.987–0.997).

**Conclusion:**

Combined PCL instabilities resulted in a significant increase in posterior tibial displacement in posterior stress radiographs. However, PSR in IR or ER was unable to differentiate between these combined instabilities. Based on our data, additional stress radiographs in rotation are unlikely to provide any diagnostic benefit in the clinical setting.

**Level of Evidence:**

There is no level of evidence as this study was an experimental laboratory study.

**Abbreviations**
ER: external rotation
ICC: intraclass correlation coefficient
IR: internal rotation
LCL: lateral collateral ligament
MCL: medial collateral ligament
NR: neutral rotation
PCL: posterior cruciate ligament
PFL: popliteofibular ligament
PLC: posterolateral complex
PMC: posteromedial complex
POL: posterior oblique ligament
PSR: posterior stress radiography
PTD: posterior tibial displacement

## INTRODUCTION

Most isolated complete or partial posterior cruciate ligament (PCL) injuries are initially treated conservatively with satisfactory results due to the inherent healing capability [26, 29]. The decision for surgical treatment is based on several factors, including patient's demand, the presence of subjective and objective instability, and the existence of combined instabilities [2, 20, 29]. If a posterior instability is identified during the clinical examination, posterior stress radiography (PSR) is generally recommended to distinguish between isolated and combined PCL instability [3, 9, 12]. The Telos device and kneeling radiography are most commonly used [9, 13], and a side‐to‐side difference of more than 10–12 mm posterior tibial displacement (PTD) is considered indicative of a combined injury, which should be evaluated for surgical treatment [8, 13, 17, 25].

To provide appropriate surgical treatment with good clinical and functional outcomes, it is essential to identify a posterolateral or posteromedial instability [1, 4, 7], as these conditions represent a significant risk factor for failure if left untreated [19, 22]. Given the nature of the instabilities, rotational posterior drawer tests can aid in detecting subtle rotational instabilities, as the posterior drawer in external rotation may be positive for posterolateral complex (PLC) instabilities and the posterior drawer in internal rotation for posteromedial complex (PMC) instabilities [28]. However, detecting these subtle differences is challenging and primarily depends on the expertise of the clinician. Therefore, a quantifiable radiographic measurement method would be beneficial.

The objective of this study was to assess the posterior tibial displacement in posterior stress radiographs for both isolated and combined (PLC and PMC) PCL deficiencies, with the foot positioned in internal rotation (IR) and external rotation (ER), compared to PTD in neutral rotation (NR). Based on clinical observations, it was hypothesized that an additional PLC injury will increase the radiographic PTD in external rotation, while an additional PMC injury will increase the radiographic PTD in internal rotation [28].

## METHODS

Six paired (*n* = 12) cadaveric leg specimens (mean age 83.3 ± 6.3 years, all female, and all Caucasian) from the anatomical department of the University of Luebeck (Germany) were included in the study. The exclusion criteria included prior surgery, high‐grade osteoarthritis, and ligamentous instability. Prior to testing, ligamentous integrity was confirmed through a clinical examination. High‐grade osteoarthritis was ruled out after testing was performed by ex‐articulating the knee joint. All 12 legs could be included in the final analysis. The specimens were stored at −20°C and thawed for 24 h at room temperature, prior to preparation and testing. Before testing, the knees were flexed 10 times to prevent tissue hysteresis.

### Test setup

**Figure 1:**
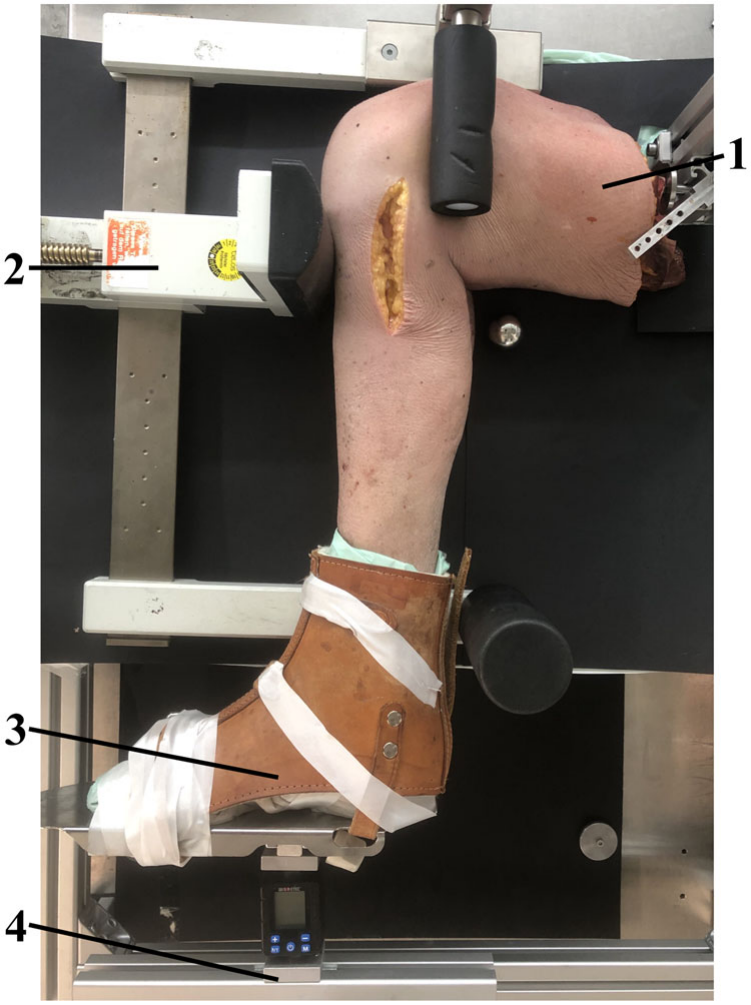
Right leg specimen in a Telos device with a modified foot apparatus (3). After rotation was performed, posterior stress was applied using the pressure device (2), by turning a grip clockwise until 15 kp was reached. Rotation was measured at the longitudinal axis of the foot apparatus (1: specimen; 2: Telos device; 3: custom‐made foot apparatus; 4: custom‐made aluminium frame to stabilize the specimen and the foot apparatus).

The specimens were mounted in a PSR device GA‐III/E (Telos GmbH). The femur was secured to a custom‐made aluminium frame via an intramedullary nail, which was fixed using two bicortical screws. The foot was secured in a neutral position in a custom‐made foot apparatus to reduce ankle laxity and to apply IR or ER. A force of 15 kp (147.1 N) was applied to the tibial tubercle in 90° knee flexion. This was repeated with the foot placed in 30° IR and ER. For each cutting sequence, this was repeated three times (Figure 1).

### Sequential cutting and testing protocol

The PCL was cut at its tibial insertion via a posteromedial approach [10]. Following the cutting of the PCL in all knee specimens (*N* = 12), six were utilized to create a combined posterolateral deficiency, while the remaining six were used for a combined posteromedial deficiency.

In the posterolateral sequence, the lateral collateral ligament (LCL) was initially cut at its mid‐substance via a small incision, followed by a scalpel being slid along the popliteus tendon to cut the popliteofibular ligament (PFL). Subsequently, the PMC was cut in accordance with the posteromedial sequence.

**Figure 2:**
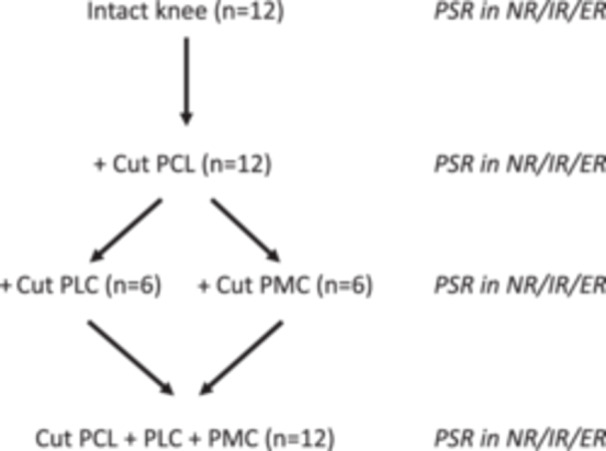
The cutting protocol. In 12 intact knees, posterior stress radiographs (PSR) for posterior tibial displacement (PTD) in neutral (NR)/internal (IR)/external (ER) rotation were performed. Then, the posterior cruciate ligament (PCL) was cut in all 12 knees, and PSR was repeated in NR/IR/ER. Then, the posterolateral complex (PLC) was cut in six knees, while in the other six knees, the posteromedial complex (PMC) was cut, and PSR was repeated in NR/IR/ER. Subsequently, the PMC or PLC corner, which was initially kept intact, was cut, creating 12 PCL + PLC + PMC deficient knees, which were again tested in NR/IR/ER.

In the posteromedial sequence, the superficial medial collateral ligament (MCL), the deep MCL, and the posterior oblique ligament (POL) were cut mid‐substance at the level of the joint line. Subsequently, the PLC was cut in accordance with the posterolateral sequence (Figure 2).

### Radiographic measurements

**Figure 3:**
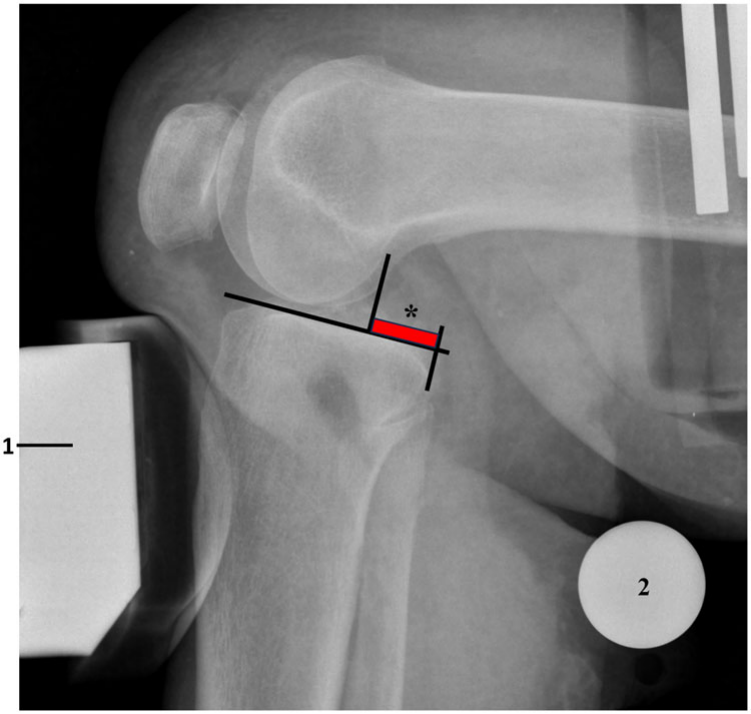
Radiographic posterior tibial displacement (PTD) according to Jacobsen [11] (1 = Pressure device of the Telos device; 2 = X‐ray reference ball with a diameter of 32 mm; *= PTD according to Jacobsen [11], which is the distance in between the two perpendicular lines).

The radiographs were performed using a mobile X‐ray source and detector (Examion X‐AQS, Examion GmbH). The source was positioned 1 m above the laterally aligned specimen (Figure 1). The detector was positioned directly beneath the custom‐made aluminium frame, simulating a clinical PSR setup. A 32 mm diameter X‐ray reference ball was used. The radiograph was repeated if the femoral/tibial overlap was deemed unsatisfactory. The radiographic images were analyzed using a DICOM Viewer (OsiriX MD v.14.0 Dicom, Pixmeo SARL). The measurements were recorded to the nearest 0.1 mm, consistent with the accuracy of the DICOM Viewer. Two experienced orthopaedic surgeons independently performed the radiographic measurements. For intraobserver reliability, rater one (T.B.) performed PTD measurements twice, while for interobserver reliability, two raters (T.B. and C.K.) each performed PTD measurements once. Radiographic measurements were performed according to the Jacobson method [11], which involved drawing a line along the posterior tibial slope onto the medial tibial plateau, followed by two perpendicular lines through the midpoint of the most posterior medial and lateral tibial and femoral condyles. The distance between these two perpendicular lines was defined as the PTD (Figure 3).

### Ethics approval

The experiments were performed with the approval of the Institutional Review Board of the University of Münster (Germany) (IRB reference number 2023‐407‐f‐S).

### Data and statistical analysis

The absolute and relative (compared to the intact state) PTD was recorded for each state and rotation, and the three measurements were averaged. Descriptive statistics, including mean and standard deviation, were calculated. The statistical analysis was performed using GraphPad Prism 10 (Version 10.0.0). A two‐way ANOVA and a mixed model with post hoc Bonferroni correction were conducted. A *p* value less than 0.05 was considered to indicate a statistically significant difference.

An a priori power analysis was conducted using G*Power‐2 software (University Düsseldorf) [5]. A two‐tailed *t* test with means (difference between two independent means, two groups) was selected in G*Power‐2 for the power analysis. Based on the means and standard deviations from prior studies [8, 27], it was assumed that a sample size of 6 would allow for the identification of changes in PTD of 4 mm, with a standard deviation of 2 mm (effect size/Cohen's *d* = 2), with 80% power, at the significance level of *p* < 0.05.

The intra‐ and interobserver reliability of the measurements was determined by calculating the intraclass correlation coefficient (ICC) for PTD in Excel (Microsoft). An ICC of 0.60–0.74 was considered good, and 0.75–1.00 was considered excellent reliability [6].

## RESULTS

**Figure 4:**
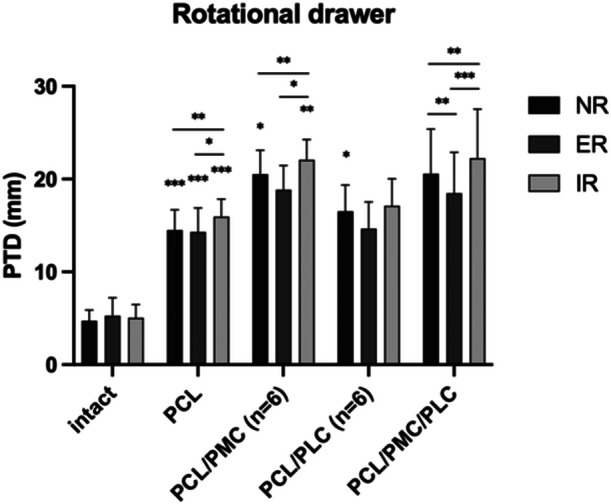
Posterior tibial displacement (PTD) measured in posterior stress radiographs after cutting the posterior cruciate ligament (PCL) and the medial (PMC) and lateral (PLC) structures. Posterior displacement was applied in neutral rotation (NR) and with the foot placed in 30° external (ER) and 30° internal (IR) tibial rotation. Statistically significant change compared to the previous state of the knee. Lines indicate statistically significant differences between rotations. *p < 0.05; **p < 0.01; ***p < 0.001.

Effect of a posterior drawer in NR on an isolated and combined PCL instability: When the PCL was cut, the radiographic PTD increased by 9.8 ± 1.8 mm (*n* = 12; *p* < 0.001) in NR, compared to the intact state. This further increased 2.4 ± 0.9 mm (*n* = 6; *p* < 0.01) with an additional PLC deficiency and 5.6 ± 2.3 mm (*n* = 6; *p* < 0.05) with an additional PMC deficiency. Cutting both the PLC and PMC further increased the radiographic PTD to 15.9 ± 4.5 mm (*n* = 12; p < 0.01) compared to the intact state of the knee (Table 1 and Figure 4 for additional details).

**Table 1:** Left column: Posterior tibial displacement (PTD) of the intact knee (mean ± SD in mm) and side‐to‐side difference of PTD of each cutting state (mean ± SD in mm) in internal (IR)/neutral (NR)/external (ER) rotation, compared to the intact state. Significance compared to the previous state of the knee. Right column: Difference of PTD between NR versus ER and IR versus NR and IR versus ER (mean ± SD in mm) for each cutting state. Significance between rotations.

Cutting state	Rotation	PTD (mm)	*p* Value	Difference between rotations	Difference of PTD (mm) between rotations	*p* Value
Intact ligaments (*n* = 12)	IR	5.1 ± 1.4	‐	NR vs. ER	0.6 ± 1.1	ns
	NR	4.7 ± 1.2	‐	IR vs. NR	0.4 ± 1.8	ns
	ER	5.3 ± 1.9	‐	IR vs. ER	0.2 ± 2.4	ns

Cutting state	Rotation	Side‐to‐side difference (PTD) (mm)	*p* Value	Difference between rotations	Difference between rotations PTD (mm)	*p* Value
Posterior cruciate ligament (PCL) (*n* = 12)	IR	10.9 ± 2.1	<0.001	NR vs. ER	0.2 ± 0.9	ns
	NR	9.8 ± 1.8	<0.001	IR vs. NR	1.4 ± 1.0	<0.01
	ER	9.0 ± 2.0	<0.001	IR vs. ER	1.6 ± 1.7	<0.05
PCL + posterolateral complex (PLC) (*n* = 6)	IR	12.6 ± 2.9	ns	NR vs. ER	1.9 ± 1.7	ns
	NR	12.2 ± 2.3	<0.05	IR vs. NR	0.6 ± 0.6	ns
	ER	9.5 ± 0.8	ns	IR vs. ER	2.5 ± 2.1	ns
PCL + posteromedial complex (PMC) (*n* = 6)	IR	16.5 ± 2.6	<0.01	NR vs. ER	1.7 ± 1.7	ns
	NR	15.4 ± 3.4	<0.05	IR vs. NR	1.6 ± 0.7	<0.01
	ER	13.4 ± 3.2	ns	IR vs. ER	3.2 ± 1.9	<0.05
PCL + PLC + PMC (*n* = 12)	IR	17.2 ± 5.5	ns	NR vs. ER	2.1 ± 1.5	<0.01
	NR	15.9 ± 4.5	ns	IR vs. NR	1.7 ± 1.1	<0.01
	ER	13.5 ± 4.5	ns	IR vs. ER	3.8 ± 2.4	<0.001

Abbreviation: ns, not significant.

### Effect of a rotational drawer on a combined PCL/PLC instability

When applying ER in the combined PCL/PLC deficient state, the PTD was 1.9 ± 1.7 mm (ns) lower compared to NR and 2.5 ± 2.1 mm (ns) lower compared to IR, which was not statistically significant (Table 1 and Figure 4 for additional details).

### Effect of a rotational drawer on a combined PCL/PMC instability

Multiple comparisons revealed that when the foot was placed in IR, radiographic PTD was 1.6 ± 0.7 mm (*p* < 0.01) higher than NR and 3.2 ± 1.9 mm (*p* < 0.05) higher than ER for the PCL/PMC deficient state compared to the PTD in NR and PTD in ER (Table 1 and Figure 4 for additional details).

### Intra‐ and Interobserver reliability

Our measurements showed excellent intra‐ and interobserver reliability. Please refer to Table 2 for additional details.

**Table 2:** Intra‐ and interobserver reliability for radiographic measurements.

Internal rotation		Neutral rotation		External rotation	
Intra	Inter	Intra	Inter	Intra	Inter
0.993	0.997	0.987	0.997	0.994	0.995

## DISCUSSION

The main finding of the present study was that, contrary to our initial hypothesis, combined posterolateral or posteromedial PCL injuries could not be accurately identified using stress radiographs in IR or ER. Although posterior displacement in IR increased by 1.6 mm compared to the NR in a simulated posteromedial injury, this small increase may not be clinically relevant because it falls within the accuracy range of previously performed PCL stress radiography studies [8, 11]. Consequently, the clinical use of stress radiographs in rotation to detect combined PCL injuries is questionable.

It is known from in vitro studies with varying testing setups that a complete PCL transection yields a pooled 10.7 mm PTD side‐to‐side difference [15]. Among these, stress radiography studies [8, 25], reported a side‐to‐side difference of 9.8 mm following isolated PCL instability. This exactly matched the radiographic PTD in NR in the present study, even though different posterior loads (200 [25] /180 N [8] vs. 15 kp (147.1 N) in the present study) and X‐ray analysis techniques (Stäubli et al. [27] vs. Jacobson [11]) were used. The present study also examined combined PCL with PLC and PMC injuries. Consistent with other stress radiography studies, combined injuries demonstrated a mean PTD of over 10 [25] or 12 [8] mm side‐to‐side difference (PLC: 12.2 mm; PMC: 15.4 mm in the present study). However, previous studies have reported a higher PTD in combined PCL and PLC deficiencies (ranging from 13.8 to 19.4 mm [8, 25]) compared to the present study. This discrepancy may be attributed to the fact that we did not resect the popliteus tendon but instead only cut the popliteofibular ligament, which may have led to a reduced PTD in rotational stress radiographs.

In contrast to the posterolateral side, posteromedial instabilities are less frequently discussed in the literature, with studies mainly assessing PTD without the use of stress radiography [21, 23]. For example, Robinson et al. [23] demonstrated only a minimal increase of 2.2 mm in PTD when cutting the PMC and applying a 150 N posterior tibial load in 5 N m of IR, although their data were limited to early flexion angles. In a study by Moslemian et al. [21], PTD did not significantly increase after cutting the POL under a 100 N posterior tibial load combined with 5 N m IR. However, they reported that after cutting the sMCL, the knee became so unstable that they could not report the kinematic data [21]. In the present study, we cut both the sMCL and POL, demonstrating a significant increase in PTD in both NR and IR, highlighting the importance of the PMC in restraining posterior tibial translation. Therefore, in a clinical setting, when observing high‐grade PTD, PMC instability should be ruled out.

The clinical relevance of this study was to evaluate the effectiveness of PSR in IR/ER to differentiate a subtle combined PCL/PLC or PCL/PMC instability. However, our findings did not provide consistent PTD values in IR or ER, that could be used to develop a reliable diagnostic algorithm for combined PCL instabilities. One possible reason for these inconsistent results is the limitation of the Jacobson method, which measures PTD using the midpoint of the posterior tibial and femoral condyles. This approach may not effectively differentiate between medial and lateral PTD, potentially leading to the observed variability in our results. Consequently, while PTD in each compartment may change with IR and ER, the overall PTD may remain unchanged with this method. In the clinical setting, performing four additional radiographs (IR and ER rotation for the ipsilateral and contralateral side) may not be justified. Instead, a more accurate diagnosis of combined PCL instabilities may be achieved by assessing the PTD in NR in combination with a thorough clinical examination.

This study had several limitations inherent to in vitro cadaveric biomechanical studies. The age of the specimens used was higher than that of most patients who experience combined instabilities. Additionally, the simulated deficiencies only mimic the injury pattern of acute time‐zero combined PCL lesions. PCL injuries are likely to heal over time [18] or present with partial lesions with varying degrees of instability, which could not be fully replicated in this test setup. Despite these limitations, the cadaveric setup was chosen to ensure accurate and repeatable simulated instabilities to evaluate our hypothesis. An in vivo study would require additional X‐rays, increasing radiographic exposure for young patients, and may not be ethically accepted. Another limitation was certainly the foot apparatus, which allowed 30° of IR or ER. This degree of rotation was greater than initially described for the external rotation posterior drawer test (15° external rotation) [17, 28]. This was necessary, as some of the rotation was lost due to the ankle laxity of the older cadaveric specimens. However, the ankle joint was positioned and stabilized in a neutral position in the foot apparatus to minimize this laxity.

A strength of the present study was the ability to achieve satisfactory alignment of the posterior femoral condyles in the laboratory setup, a challenge that is not always met in the clinical setting using PCL stress radiography. Therefore, it is likely that our ICC values were higher compared to those reported in clinical studies [14, 16, 24].

## CONCLUSION

Combined PCL instabilities resulted in a significant increase in posterior tibial displacement in posterior stress radiographs. However, posterior stress radiography in internal‐ or external rotation was unable to differentiate between these combined instabilities. Based on our data, additional stress radiographs in rotation are unlikely to provide any diagnostic benefit in the clinical setting.

## AUTHOR CONTRIBUTIONS

**Thorben Briese**: Conception and design, testing and data acquisition, statistical analysis and writing. **Romy Riemer**: Testing and data acquisition, and writing. **Adrian Deichsel**: Internal review and statistical analysis. **Christian Peez**: Internal review and statistical analysis. **Elmar Herbst**: Internal review. **Johannes Glasbrenner**: Internal review. **Michael Raschke**: Internal review. **Christoph Kittl**: Conception and design, testing and data acquisition, statistical analysis and writing.

## CONFLICT OF INTEREST STATEMENT

Elmar Herbst is a Deputy Editor‐in‐Chief of Knee Surgery, Sports Traumatology and Arthroscopy (KSSTA). Adrian Deichsel is the Web Editor of KSSTA. All other authors have no relevant financial or non‐financial interests to disclose.

## ETHICS STATEMENT

The specimens were dissected and biomechanically tested under the approval of the Institutional Ethics Committee of the University of Muenster (File number 2023‐407‐f‐S).

## Data Availability

Data are available from the corresponding author upon reasonable request.

## References

[1] Arciero, R.A. (2010) Pearls and pitfalls in the management of the chronic multiple ligament‐injured knee. Operative Techniques in Sports Medicine, 18, 250–266. Available from: 10.1053/j.otsm.2010.08.001

[2] Chahla, J. , Kunze, K.N. , LaPrade, R.F. , Getgood, A. , Cohen, M. , Gelber, P. et al. (2021) The posteromedial corner of the knee: an international expert consensus statement on diagnosis, classification, treatment, and rehabilitation. Knee Surgery, Sports Traumatology, Arthroscopy, 29, 2976–2986. Available from: 10.1007/s00167-020-06336-3 PMC7586411 33104867

[3] Chahla, J. , von Bormann, R. , Engebretsen, L. & LaPrade, R.F. (2016) Anatomic posterior cruciate ligament reconstruction: state of the art. Journal of ISAKOS, 1, 292–302. Available from: 10.1136/jisakos-2016-000078

[4] Drenck, T.C. , Frings, J. , Preiss, A. , Muellner, M. , Akoto, R. , Alm, L. et al. (2022) The treatment of posterolateral knee instability with combined arthroscopic popliteus bypass and PCL reconstruction provides good‐to‐excellent clinical results in the mid‐term follow‐up. Knee Surgery, Sports Traumatology, Arthroscopy, 30, 1414–1422. Available from: 10.1007/s00167-021-06590-z 34059968

[5] Faul, F. , Erdfelder, E. , Buchner, A. & Lang, A.‐G. (2009) Statistical power analyses using G*Power 3.1: tests for correlation and regression analyses. Behavior Research Methods, 41, 1149–1160. Available from: 10.3758/BRM.41.4.1149 19897823

[6] Fleiss, J. (1986) Reliability of measurement. The design and analysis of clinical experiments. New York: Wiley, pp. 1–32.

[7] Fortier, L.M. , Knapik, D.M. , Condon, J.J. , DeWald, D. , Khan, Z. , Kerzner, B. et al. (2023) Higher success rate observed in reconstruction techniques of acute posterolateral corner knee injuries as compared to repair: an updated systematic review. Knee Surgery, Sports Traumatology, Arthroscopy, 31, 5565–5578. Available from: 10.1007/s00167-023-07582-x 37848567

[8] Garavaglia, G. , Lubbeke, A. , Dubois‐Ferrière, V. , Suva, D. , Fritschy, D. & Menetrey, J. (2007) Accuracy of stress radiography techniques in grading isolated and combined posterior knee injuries: a cadaveric study. The American Journal of Sports Medicine, 35, 2051–2056. Available from: 10.1177/0363546507306466 17885222

[9] Guth, J.J. , Brophy, R.H. , Matava, M.J. , Steinmetz, R.G. & Smith, M.V. (2022) Stress radiography is a reliable method to quantify posterior cruciate ligament insufficiency: a systematic review. Arthroscopy, Sports Medicine, and Rehabilitation, 4, e1851–e1860. Available from: 10.1016/j.asmr.2022.05.013 36312726 PMC9596873

[10] Hooper, P.O. , Bevan, P.J. , Silko, C. & Farrow, L.D. (2018) A posterior approach to open reduction and internal fixation of displaced posterior cruciate ligament tibial osseous avulsions. JBJS Essential Surgical Techniques, 8, e6. Available from: 10.2106/JBJS.ST.17.00044 30233978 PMC6143298

[11] Jacobsen, K. (1976) Stress radiographical measurement of the anteroposterior, medial and lateral stability of the knee joint. Acta Orthopaedica Scandinavica, 47, 335–344. Available from: 10.3109/17453677608992002 952223

[12] James, E.W. , Williams, B.T. & LaPrade, R.F. (2014) Stress radiography for the diagnosis of knee ligament injuries: a systematic review. Clinical Orthopaedics & Related Research, 472, 2644–2657. Available from: 10.1007/s11999-014-3470-8 24504647 PMC4117881

[13] Jung, T.M. , Reinhardt, C. , Scheffler, S.U. & Weiler, A. (2006) Stress radiography to measure posterior cruciate ligament insufficiency: a comparison of five different techniques. Knee Surgery, Sports Traumatology, Arthroscopy, 14, 1116–1121. Available from: 10.1007/s00167-006-0137-3 16799824

[14] Kim, S.G. , Kim, S.H. , Choi, W.S. & Bae, J.H. (2019) Supine lateral radiographs at 90° of knee flexion have a similar diagnostic accuracy for chronic posterior cruciate ligament injuries as stress radiographs. Knee Surgery, Sports Traumatology, Arthroscopy, 27, 2433–2439. Available from: 10.1007/s00167-018-5228-4 30361755

[15] Kowalczuk, M. , Leblanc, M.C. , Rothrauff, B.B. , Debski, R.E. , Musahl, V. , Simunovic, N. et al. (2015) Posterior tibial translation resulting from the posterior drawer manoeuver in cadaveric knee specimens: a systematic review. Knee Surgery, Sports Traumatology, Arthroscopy, 23, 2974–2982. Available from: 10.1007/s00167-015-3584-x 25837228

[16] Lee, Y.S. , Han, S.H. , Jo, J. , Kwak, K. , Nha, K.W. & Kim, J.H. (2011) Comparison of 5 different methods for measuring stress radiographs to improve reproducibility during the evaluation of knee instability. The American Journal of Sports Medicine, 39, 1275–1281. Available from: 10.1177/0363546510396182 21350067

[17] Levy, B.A. , Stuart, M.J. & Whelan, D.B. (2010) Posterolateral instability of the knee: evaluation, treatment, results. Sports medicine and arthroscopy review, 18, 254–262. Available from: 10.1097/JSA.0b013e3181f88527 21079505

[18] Mariani, P.P. , Margheritini, F. , Christel, P. & Bellelli, A. (2005) Evaluation of posterior cruciate ligament healing: a study using magnetic resonance imaging and stress radiography. Arthroscopy: The Journal of Arthroscopic & Related Surgery, 21, 1354–1361. Available from: 10.1016/j.arthro.2005.07.028 16325087

[19] Miura, S. , Iwasaki, K. , Kondo, E. , Endo, K. , Matsubara, S. , Matsuoka, M. et al. (2022) Stress on the posteromedial region of the proximal tibia increased over time after anterior cruciate ligament injury. Knee Surgery, Sports Traumatology, Arthroscopy, 30, 1744–1751. Available from: 10.1007/s00167-021-06731-4 34505928

[20] Montgomery, S.R. , Johnson, J.S. , McAllister, D.R. & Petrigliano, F.A. (2013) Surgical management of PCL injuries: indications, techniques, and outcomes. Current Reviews in Musculoskeletal Medicine, 6, 115–123. Available from: 10.1007/s12178-013-9162-2 23430587 PMC3702782

[21] Moslemian, A. , Arakgi, M.E. , Roessler, P.P. , Sidhu, R.S. , Degen, R.M. , Willing, R. et al. (2021) The medial structures of the knee have a significant contribution to posteromedial rotational laxity control in the PCL‐deficient knee. Knee Surgery, Sports Traumatology, Arthroscopy, 29, 4172–4181. Available from: 10.1007/s00167-021-06483-1 33677624

[22] Pizza, N. , Di Paolo, S. , Grassi, A. , Pagano, A. , Viotto, M. , Dal Fabbro, G. et al. (2023) Good long‐term patients reported outcomes, return‐to‐work and return‐to‐sport rate and survivorship after posterior cruciate ligament (PCL)‐based multiligament knee injuries (MLKI) with posteromedial corner tears as significant risk factor for failure. Knee Surgery, Sports Traumatology, Arthroscopy, 31, 5018–5024. Available from: 10.1007/s00167-023-07547-0 PMC10598146 37668614

[23] Robinson, J.R. , Bull, A.M.J. , deW. Thomas, R.R. & Amis, A.A. (2006) The role of the medial collateral ligament and posteromedial capsule in controlling knee laxity. The American Journal of Sports Medicine, 34, 1815–1823. Available from: 10.1177/0363546506289433 16816148

[24] Ryu, D.J. , Kwon, K.B. , Jung, E.Y. , Lee, S.S. , Kim, J.H. , Jang, M.C. et al. (2021) Clinically reliable knee flexion angle measured on stress radiography for quantifying posterior instability in posterior cruciate ligament injury. Orthopaedic Journal of Sports Medicine, 9, 2325967121989252. Available from: 10.1177/2325967121989252 34104655 PMC8172336

[25] Sekiya, J.K. , Whiddon, D.R. , Zehms, C.T. & Miller, M.D. (2008) A clinically relevant assessment of posterior cruciate ligament and posterolateral corner injuries. Evaluation of isolated and combined deficiency. The Journal of Bone and Joint Surgery‐American Volume, 90, 1621–1627. Available from: 10.2106/JBJS.G.01365 18676890

[26] Shelbourne, K.D. , Clark, M. & Gray, T. (2013) Minimum 10‐year follow‐up of patients after an acute, isolated posterior cruciate ligament injury treated nonoperatively. The American Journal of Sports Medicine, 41, 1526–1533. Available from: 10.1177/0363546513486771 23652263

[27] Staubli, H. & Jakob, R. (1990) Posterior instability of the knee near extension. A clinical and stress radiographic analysis of acute injuries of the posterior cruciate ligament. The Journal of Bone and Joint Surgery. British Volume, 72, 225–230. Available from: 10.1302/0301-620X.72B2.2312560 2312560

[28] Swinford, S.T. , LaPrade, R. , Engebretsen, L. , Cohen, M. & Safran, M. (2020) Biomechanics and physical examination of the posteromedial and posterolateral knee: state of the art. Journal of ISAKOS, 5, 378–388. Available from: 10.1136/jisakos-2018-000221

[29] Winkler, P.W. , Zsidai, B. , Wagala, N.N. , Hughes, J.D. , Horvath, A. , Senorski, E.H. et al. (2021) Evolving evidence in the treatment of primary and recurrent posterior cruciate ligament injuries, part 2: surgical techniques, outcomes and rehabilitation. Knee Surgery, Sports Traumatology, Arthroscopy, 29, 682–693. Available from: 10.1007/s00167-020-06337-2 PMC7917042 33125531

